# Electromagnetically Induced Transparency (EIT) Like Transmission Based on 3 × 3 Cascaded Multimode Interference Resonators

**DOI:** 10.3390/mi9080417

**Published:** 2018-08-09

**Authors:** Trung-Thanh Le

**Affiliations:** International School (VNU-IS), Vietnam National University (VNU), Hanoi 1000, Vietnam; thanh.le@vnu.edu.vn; Tel.: +84-985-848-193

**Keywords:** optical microring resonator, electromagnetically induced transparency (EIT), multimode interference (MMI), transfer matrix method (TMM), finite difference time difference (FDTD), beam propagation method (BPM)

## Abstract

We propose a method for generating the electromagnetically induced transparency (EIT) like-transmission by using microring resonator based on cascaded 3 × 3 multimode interference (MMI) structures. Based on the Fano resonance unit created from a 3 × 3 MMI coupler with a feedback waveguide, two schemes of two coupled Fano resonator unit (FRU) are investigated to generate the EIT like transmission. The theoretical and numerical analysis based on the coupled mode theory and transfer matrix is used for the designs. Our proposed structure has advantages of compactness and ease of fabrication. We use silicon waveguide for the design of the whole device so it is compatible with the existing Complementary Metal-Oxide-Semiconductor (CMOS) circuitry foundry. The fabrication tolerance and design parameters are also investigated in this study.

## 1. Introduction

The electromagnetically induced transparency (EIT) effect is a nonlinear effect found in the interaction process between light and material. The EIT effect has been intensively investigated in recent years [1,2]. The EIT has wide applications such as in quantum information [3], lasing without inversion [4], optical delay, slow light [5], nonlinearity enhancement [6] and precise spectroscopy [7], pushing frontiers in quantum mechanics and photonics and sensing technology [8]. In order to create the EIT effect, there are some suggested approaches.

There is a significant benefit to determine the optical EIT like transmissions with high modulation depth, which is defined by the difference in intensities between the peak and the dip at resonant wavelengths. The EIT was first observed in atomic media [2]. Then, the EIT-like effects were found in optical coupled resonant systems [9,10,11], mechanics, electrical circuits [12], plasmonics, metamaterials [7,13] and hybrid configurations [14]. In the coupled resonant systems, the basic underlying physical principle is the interference of fields instead of the probability of amplitudes, as in a three-level atomic system [15,16]. Most of the proposed structures so far for the optical EIT generation use metal-insulator-metal (MIM) plasmonic waveguide resonators [17,18], array of fiber optic resonators [19], microspheres [20], metallic arrays of asymmetric dual stripes [21], heptamer-hole array [22], plasmonic nanoring pentamers [23] and microtoroid resonator coupled system [24]. For these systems, the fiber beam splitters, directional couplers or MIM plasmonic waveguide must be used. As a result, such structures bring large size, the complexity of the fabrication process to control exactly the coupling ratios of the directional couplers and sensitivity to fabrication tolerance.

The transparency window of the EIT is caused by reduced absorption, due to the quantum destructive interference between the transitions from the two dressed states, into a common energy level. Similarly, the EIT-like effect generated by optical resonators work by the means of coherent interference between the resonating modes which produce optical transparency inside the absorption window [25]. Compared to the EIT in atomic systems, the analogue of electromagnetically induced transparency with optical resonators based on directional couplers has many remarkable advantages such as simpler structure, smaller device size and easier design. However, due to the small size of these structures, it is challenging to detune optical resonator for controlling the resonant interaction between the two optical pathways and controlling the coupling ratio of the directional couplers [26].

In the literature, only 2 × 2 directional coupler was used for microring resonator based on the EIT effects [25]. However, such structure is very sensitive to the fabrication. It has a large size and requires a complicated fabrication process. It was shown that the integration of multimode interference (MMI) and resonators can provide new physical characteristics. By using the MMIs, we can overcome the disadvantages of devices based on directional couplers such as compactness, ease of fabrication and large fabrication tolerance [27]. One of such structures is a 3 × 3 MMI based microring resonator. We have proposed for the first time microring resonator structures based on 3 × 3 and 4 × 4 MMI couplers for Fano resonance generation [28,29,30]. In this study, we further develop new structures based on only cascaded 3 × 3 multimode interference coupler based microring resonators to produce the EIT resonance like transmissions. The proposed device is analyzed and optimized using the transfer matrix method, the beam propagation method (BPM) and finite difference time difference (FDTD) [31]. A description of the theory behind the use of multimode structures to achieve the FRU and EIT effect is presented in Section 2 and Section 3. A brief summary of the results of this research is given in Section 4.

## 2. Single Fano Resonance Unit (FRU)

**Figure 1:**
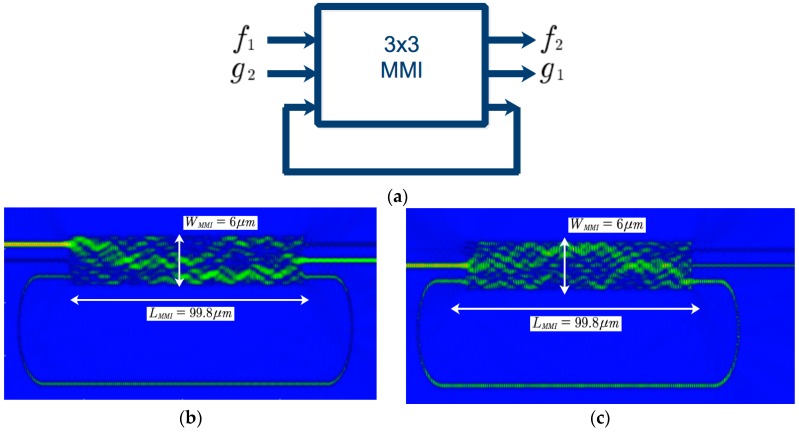
(**a**) Fano resonance unit created by 3 × 3 MMI (multimode interference) based resonator (**b**) FDTD simulation for input 1 and (**c**) finite difference time difference (FDTD) simulation for input 2.

Fano resonance can be created by many approaches such as integrated waveguide-coupled microcavities [32,33], prism-coupled square micro-pillar resonators, multimode tapered fiber coupled micro-spheres and Mach Zehnder interferometer (MZI) coupled micro-cavities [34], plasmonic waveguide structure [35,36]. We have proposed integrated photonic circuits for realizing Fano resonance based on 3 × 3 MMI and 4 × 4 MMI microring resonator [29,37]. Figure 1a shows a scheme for Fano resonance unit (FRU) based on only one 3 × 3 MMI coupler with a feedback waveguide. Figure 1b,c shows the FDTD simulation for the FRU with input signal presented at input ports 1 and 2, respectively.

In the time domain, the Fano resonance system created by 3 × 3 MMI coupler based microring resonator in Figure 1 can be expressed by the coupled mode equations [38]

(1) $$\frac{da_{n}}{dt}=[j(\omega_{0}+\delta \omega_{n})-\frac{1}{\tau }]a_{n}+df_{n}+dg_{n+1}$$

(2) $$g_{n}=\exp(j\phi )f_{n}+da_{n}$$

where *n* = 1, 2 and $d=j\exp(j\phi /2)/\sqrt{\tau }$; ϕ is the phase of the resonator depending on the feedback waveguide, $\delta \omega_{n}$ is the nonlinear phase shift, $a_{n}$ is the amplitude in the resonator mode; $f_{n}$, $g_{n}$ are the complex amplitudes at input and output ports; $\omega_{0}$ and τ are resonant frequency and lifetime of the resonator.

In the frequency domain, the 3 × 3 MMI coupler can be described by a transfer matrix $M={\left[m_{\mathrm{ij}}\right]}_{3\times 3}$ which describes the relationships between the input and output complex amplitudes (fields) of the coupler [39]. The length of the MMI coupler is to be $L_{\mathrm{MMI}}=L_{\pi }$, $L_{\pi }$ is the beat length of the MMI coupler. The relationship between the output complex amplitudes $b_{j}(j=1,\;2,\;3)$ and the input complex amplitudes $a_{i}(i=1,\;2,\;3)$ of the coupler can be expressed by [39]

(3) $$\left(\begin{array}{c} b_{1}\\ b_{2}\\ b_{3} \end{array}\right)=\frac{1}{\sqrt{3}}\left(\begin{array}{ccc} -e^{-j2\pi /3} & e^{-j2\pi /3} & -1\\ e^{-j2\pi /3} & -1 & e^{-j2\pi /3}\\ -1 & e^{-j2\pi /3} & -e^{-j2\pi /3} \end{array}\right)\left(\begin{array}{c} a_{1}\\ a_{2}\\ a_{3} \end{array}\right)=M\left(\begin{array}{c} a_{1}\\ a_{2}\\ a_{3} \end{array}\right)$$

The complex amplitudes at output ports 1 and 2 of the first microring resonator of Figure 1 are given by

(4) $$b_{1}{=(m}_{11}+\frac{m_{13}m_{31}\mathrm{aexp}(\mathrm{jq})}{{1-m}_{33}\mathrm{aexp}(\mathrm{jq})}{)a}_{1}{+(m}_{12}+\frac{m_{13}m_{32}\mathrm{aexp}(\mathrm{jq})}{{1-m}_{33}\mathrm{aexp}(\mathrm{jq})}{)a}_{2}$$

(5) $$b_{2}{=(m}_{21}+\frac{m_{23}m_{31}\mathrm{aexp}(\mathrm{jq})}{{1-m}_{33}\mathrm{aexp}(\mathrm{jq})}{)a}_{1}{+(m}_{22}+\frac{m_{23}m_{32}\mathrm{aexp}(\mathrm{jq})}{{1-m}_{33}\mathrm{aexp}(\mathrm{jq})}{)a}_{2}$$

where $\alpha =\exp(-\alpha_{0}L)$ is the transmission loss along the ring waveguide, L is the length of the feedback waveguide and $\alpha_{0}$ (dB/cm) is the loss coefficient in the core of the optical waveguide; $\theta =\beta_{0}L$ is the phase accumulated over the racetrack waveguide, where $\beta_{0}=2\pi n_{\mathrm{eff}}/\lambda$ and $n_{\mathrm{eff}}$ is the effective refractive index, λ is the wavelength.

In this study, we use silicon waveguide for the design, where $SiO_{2}$ ($n_{SiO_{2}}$ = 1.46) is used as the upper cladding material. The parameters used in the designs are as follows: the waveguide has a standard silicon thickness of $h_{co}=220\;\mathrm{nm}$ and access waveguide widths are $W_{a}=500\;\mathrm{nm}$ for single mode operation. It is assumed that the designs are for the transverse electric (TE) polarization at a central optical wavelength λ=1550 nm. In this study, we use the three dimensional beam propagation method (3D-BPM) and Finite Difference Time Domain (FDTD) to design the whole structure [40].

**Figure 2:**
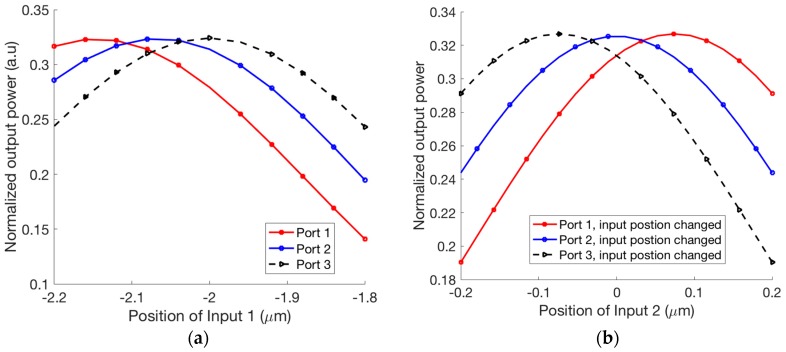
Normalized output powers for different positions of (**a**) input port 1 and (**b**) input port 2.

Firstly, we optimize the position of the access waveguide ports of the 3 × 3 MMI coupler to determine the proper matrix of the 3 × 3 MMI coupler expressed by Equation (3). The normalized output powers at output ports of the 3 × 3 MMI varying with the location of input port 1 are shown in Figure 2a. Figure 2b shows the normalized output powers at output ports for different locations of input port 2. Here, the width and length of the MMI coupler are optimized by the BPM simulations to be $W_{\mathrm{MMI}}=6\;\mu m{\;\mathrm{and}\;L}_{\mathrm{MMI}}=99.8\;\mu m$. As a result, the optimal positions of the input ports 1 and 3 are $p_{1,3}=\mp \;2.05\;\mu m$, respectively.

**Figure 3:**
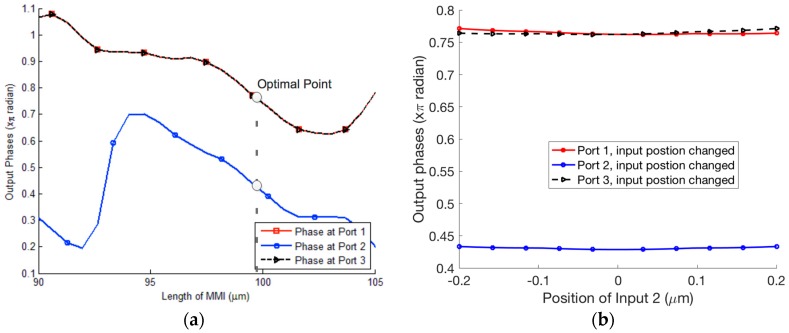
The sensitivity of phases in output ports to (**a**) MMI (multimode interference) length and (**b**) position of input 2.

The phase sensitivity of the output signals to the length variation of the 3 × 3 MMI coupler based microring resonators is particularly important to device performance. We use the BPM to investigate the effect of the MMI length on the phase sensitivity. Figure 3a shows the phases at output ports of the 3 × 3 MMI coupler at different MMI lengths. We see that a change of ±10 nm in MMI length causes a change of 4.7 × 10^−4^ π (rad) in output phases. For the existing CMOS circuitry with a fabrication error of ±5 nm [41], this is feasible and has a very large fabrication tolerance. Similarly, we consider the effect of the positions of input waveguides on the phase sensitivity as shown in Figure 3b. For a fabrication tolerance in the MMI length of ±50 nm, the fluctuation in phases is nearly unchanged.

## 3. Coupled Fano Resonances and Generation of the EIT Effect

**Figure 4:**
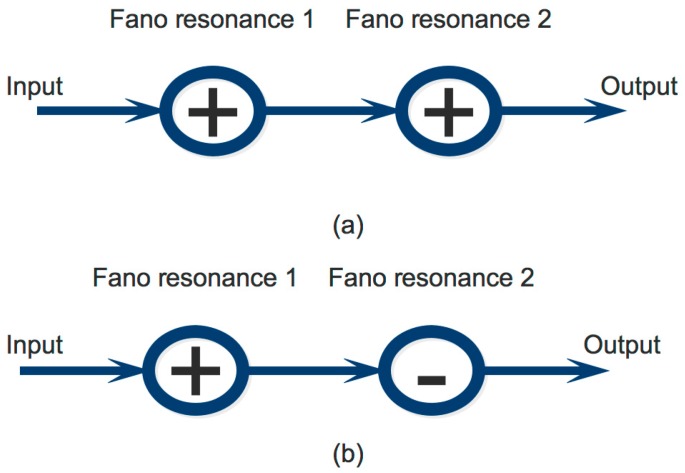
Schemes of Coupled Fano resonances (**a**) bar connect and (**b**) cross connect.

The schemes for coupled Fano resonances to generate the EIT effect is modeled in Figure 4, where single Fano resonance 1 and Fano resonance 2 of Figure 4a is exactly the same and Fano resonance 1 and Fano resonance 2 of Figure 4b is different with an exchange of input ports. We show that by cascading two Fano resonances as shown in Figure 4, the EIT effects can be created. The exchange unit can be realized by using only one 2 × 2 MMI coupler as shown in reference [42].

**Figure 5:**
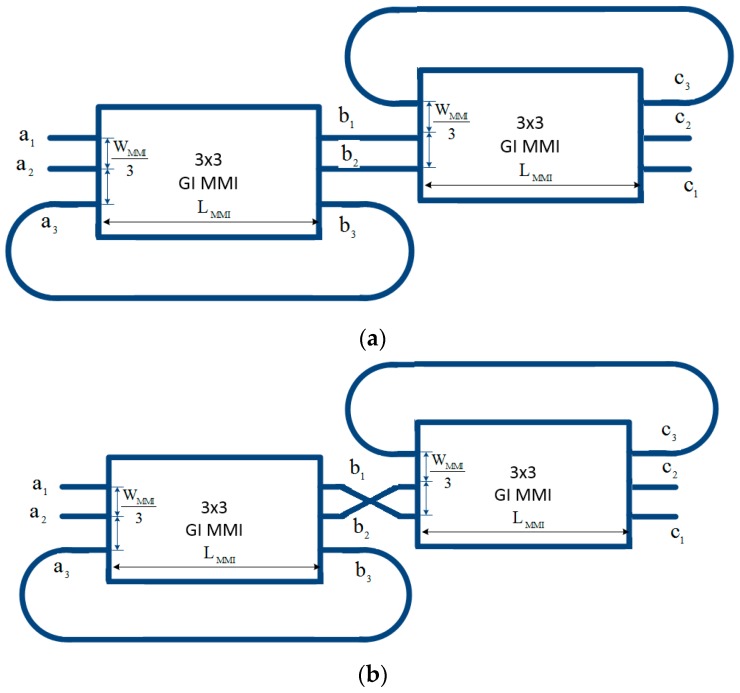
Coupled Fano resonances based on microring resonators based on 3 × 3 cascaded MMI couplers (**a**) cross without cross-connect and (**b**) bar with cross-connect made from 2 × 2 MMI coupler [42].

In our case, the Fano resonance 1 and 2 are the FRU created by 3 × 3 MMI coupler based microring resonator as shown in Figure 1. The first schemes of Figure 4a can be made as shown in Figure 5a and the second scheme of Figure 4b can be made as shown in Figure 5b.

By using analytical analysis, the transmissions at the output ports of Figure 5a, for the input signal presented at input port 1 ($a_{2}=0$) are expressed by

(6) $$T_{1}={\left|\left(m_{11}+\frac{m_{13}m_{31}\alpha e^{j\theta }}{1-m_{33}\alpha e^{j\theta }})(m_{11}+\frac{m_{13}m_{31}\alpha e^{j\theta }}{1-m_{33}\alpha e^{j\theta }})+(m_{12}+\frac{m_{13}m_{32}\alpha e^{j\theta }}{1-m_{33}\alpha e^{j\theta }})(m_{21}+\frac{m_{23}m_{31}\alpha e^{j\theta }}{1-m_{33}\alpha e^{j\theta }}\right)\right|}^{2}$$

(7) $$T_{2}={\left|\left(m_{21}+\frac{m_{23}m_{31}\alpha e^{j\theta }}{1-m_{33}\alpha e^{j\theta }})(m_{11}+\frac{m_{13}m_{31}\alpha e^{j\theta }}{1-m_{33}\alpha e^{j\theta }})+(m_{22}+\frac{m_{23}m_{32}\alpha e^{j\theta }}{1-m_{33}\alpha e^{j\theta }})(m_{21}+\frac{m_{23}m_{31}\alpha e^{j\theta }}{1-m_{33}\alpha e^{j\theta }}\right)\right|}^{2}$$

The transmissions at these output ports of Figure 5b, for the input signal presented at input port 1 ($a_{2}=0$) are

(8) $$T_{1}^{\prime }={\left|\left(m_{11}+\frac{m_{13}m_{31}\alpha e^{j\theta }}{1-m_{33}\alpha e^{j\theta }})(m_{22}+\frac{m_{23}m_{32}\alpha e^{j\theta }}{1-m_{33}\alpha e^{j\theta }})+(m_{12}+\frac{m_{13}m_{32}\alpha e^{j\theta }}{1-m_{33}\alpha e^{j\theta }})(m_{12}+\frac{m_{13}m_{32}\alpha e^{j\theta }}{1-m_{33}\alpha e^{j\theta }}\right)\right|}^{2}$$

(9) $$T_{2}^{\prime }={\left|\left(m_{21}+\frac{m_{23}m_{31}\alpha e^{j\theta }}{1-m_{33}\alpha e^{j\theta }})(m_{22}+\frac{m_{23}m_{32}\alpha e^{j\theta }}{1-m_{33}\alpha e^{j\theta }})+(m_{22}+\frac{m_{23}m_{32}\alpha e^{j\theta }}{1-m_{33}\alpha e^{j\theta }})(m_{12}+\frac{m_{13}m_{32}\alpha e^{j\theta }}{1-m_{33}\alpha e^{j\theta }}\right)\right|}^{2}$$

**Figure 6:**
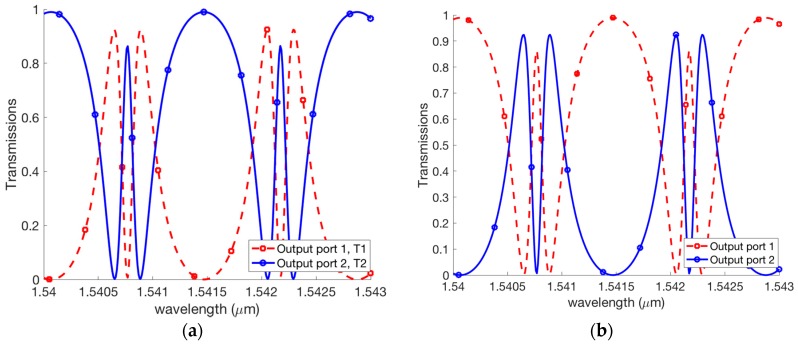
Transmissions of the coupled Fano resonances for Figure 5a with input signal is presented at (**a**) input port 1 and (**b**) input port 2.

For our design, the silicon waveguide is used. The effective refractive index calculated by the FDM (Finite Difference Method) is to be $n_{eff}=2.416299$ for the TE polarization. It assumed that the loss coefficient of the silicon waveguide is α=0.98 [43], the length of the feedback waveguide is $L_{R}=700\;\mu m$ [25]. For the first scheme of Figure 5a, the EIT effects shown in Figure 6a can be generated at output ports 1 and 2 while the input signal is at the input port 1. Figure 6b shows the EIT effects are also created at output ports 1 and 2 while the input signal is presented at input port 2. We see that the modulation depth of 80% for these EIT like transmissions have been achieved. As a result, our structure can generate both the W-shape and M-shape transmissions. Such shapes can be useful for optical switching, fast and slow light and sensing applications.

**Figure 7:**
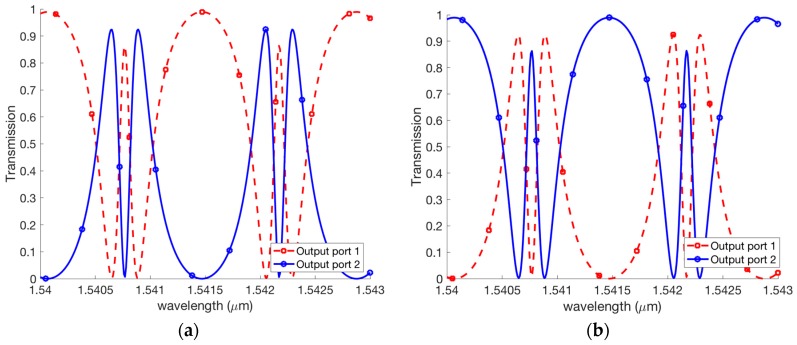
Transmissions of the coupled Fano resonances for Figure 5b with input signal is presented at (**a**) input port 1 and (**b**) input port 2.

For the second scheme of Figure 5b, the EIT effects shown in Figure 7a can be generated at output port 1 and port 2 while input signal is at input port 1. Figure 7b shows the EIT effects are also created at output ports 1 and 2 while the input signal is presented at input port 2.

**Figure 8:**
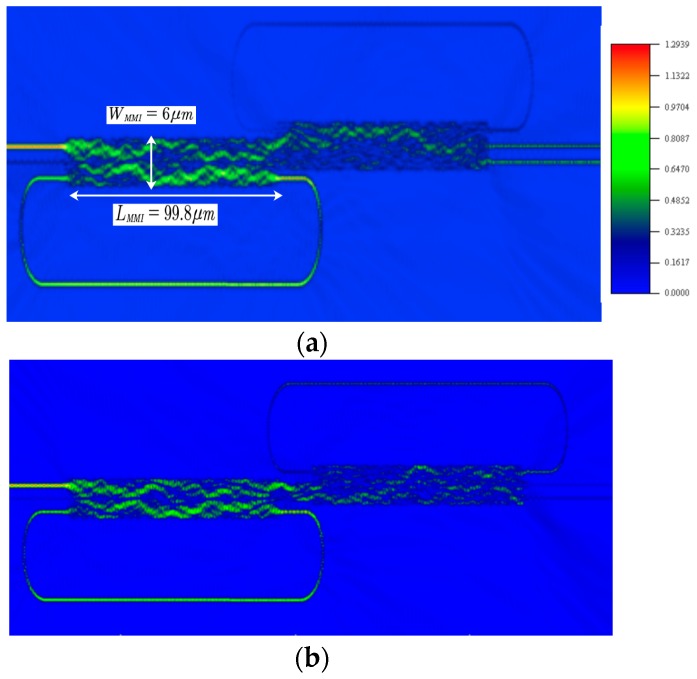
FDTD simulations for input signal at input port 1 for the EIT scheme of Figure 5a,b at wavelength λ=1550 nm.

**Figure 9:**
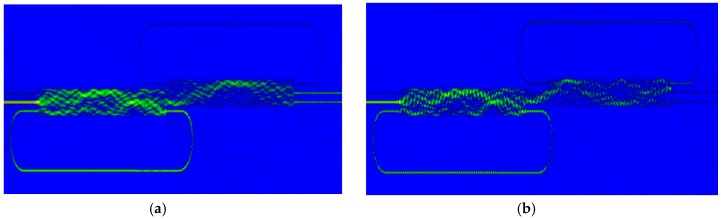
FDTD simulations for input signal at input port 2 for the EIT scheme of Figure 5a,b λ=1550 nm.

In order to verify our proposed analytical theory, we use the FDTD for accurate predictions of the device’s working principle. Figure 8 shows the FDTD simulations for the device of Figure 5a,b for input signal at port 1, respectively. Figure 9 shows the FDTD of Figure 5a,b for input signal at port 2. In our FDTD simulations, we take into account of the refractive index of silicon material calculated by using the Sellmeier equation [44,45]:

(10) $$n^{2}(\lambda )=\varepsilon +\frac{A}{\lambda^{2}}+\frac{B\lambda_{1}^{2}}{\lambda^{2}-\lambda_{1}^{2}}$$

where $\varepsilon =11.6858,\;A=0.939816\;mm^{2},\;B=8.10461\times 10^{-3}$ and $\lambda_{1}^{}=1.1071\;mm$.

In our FDTD simulations, a Gaussian light pulse of 15 fs pulse width is launched from the input to investigate the transmission characteristics of the device. The grid sizes Δx=Δy=5 nm and Δz=10 nm are chosen [46].

**Figure 10:**
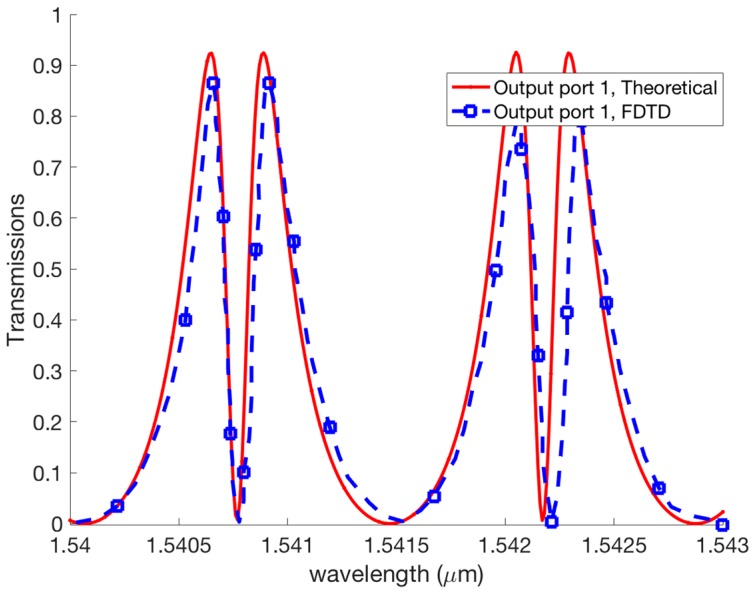
Comparison of theoretical and FDTD simulations.

For the purpose of comparing the theoretical and FDTD analysis, we investigate a comparison of the EIT like transmission effect between the theory and FDTD simulations. It is shown that the FDTD simulation has a good agreement with our theoretical analysis as presented in Figure 10.

## 4. Conclusions

We have presented a new method for the generation of the EIT effect based on coupled 3 × 3 MMI based microring resonators. Both of the M-shape and W-shape like transmissions are created. The device based on silicon waveguide, that is compatible with the existing CMOS circuitry, has been optimally designed. Our FDTD simulations show a good agreement with the theoretical analysis based on the transfer matrix method. The EIT effect can be determined based on these structures with advantages of ease of fabrication and large fabrication tolerance.
